# Road traffic noise exposure and its impact on health: evidence from animal and human studies—chronic stress, inflammation, and oxidative stress as key components of the complex downstream pathway underlying noise-induced non-auditory health effects

**DOI:** 10.1007/s11356-024-33973-9

**Published:** 2024-07-08

**Authors:** Ane Arregi, Oscar Vegas, Aitana Lertxundi, Ana Silva, Isabel Ferreira, Ainhoa Bereziartua, Maria Teresa Cruz, Nerea Lertxundi

**Affiliations:** 1 Faculty of Psychology, University of the Basque Country (UPV/EHU), 20008 San Sebastian, Spain; 2 Environmental Epidemiology and Child Development Group, Biogipuzkoa Health Research Institute, Paseo Doctor Begiristain S/N, 20014 San Sebastian, Spain; 3 Spanish Consortium for Research On Epidemiology and Public Health (CIBERESP), Instituto de Salud Carlos III, C/Monforte de Lemos 3-5, 28029 Madrid, Spain; 4 Department of Preventive Medicine and Public Health, Faculty of Medicine, University of the Basque Country (UPV/EHU), 48940 Leioa, Spain; 5 Center for Neuroscience and Cell Biology and Institute for Biomedical Imaging and Life Sciences, University of Coimbra, 3000-548 Coimbra, Portugal; 6 Center for Innovative Biomedicine and Biotechnology (CIBB), University of Coimbra, Coimbra, Portugal; 7 Faculty of Pharmacy, University of Coimbra, 3000-548 Coimbra, Portugal

**Keywords:** Road traffic noise, Environmental stressor, Inflammation, Oxidative stress, Non-auditory health effects

## Abstract

In heavily urbanized world saturated with environmental pollutants, road traffic noise stands out as a significant factor contributing to widespread public health issues. It contributes in the development of a diverse range of non-communicable diseases, such as cardiovascular diseases, metabolic dysregulation, cognitive impairment, and neurodegenerative disorders. Although the exact mechanisms behind these non-auditory health effects remain unclear, the noise reaction model centres on the stress response to noise. When exposed to noise, the body activates the hypothalamic–pituitary–adrenal axis and the sympathetic nervous system, leading to the secretion of stress hormones like catecholamines and cortisol. Prolonged exposure to noise-induced stress results in chronic inflammation and oxidative stress. This review underscores the role of inflammation and oxidative stress in the progression of noise-induced vascular dysfunction, disruption of the circadian rhythm, accelerated aging, neuroinflammation, and changes in microbiome. Additionally, our focus is on understanding the interconnected nature of these health outcomes: These interconnected factors create a cascade effect, contributing to the accumulation of multiple risk factors that ultimately lead to severe adverse health effects.

## Introduction

Currently, 55% of the global population lives in cities and this number projected to rise to 68% by 2050. Europe, in particular, has a higher urban population, with 74% of Europeans currently living in urban areas (United Nations 2018). In this scenario, the establishment of sustainable and healthy urban environments is crucial. Within the exposome, which includes the sum of all environmental contributions during the life course, involving external factors, behavioural factors, lifestyle factors, and biological responses (Daiber et al. 2019), environmental noise stands as the second most serious environmental risk factor in Europe, with air pollution being the primary contributor (European Environment Agency 2020).

The World Health Organization (WHO) defines it as noise created from all sources, except workplace noise (WHO 2018). However, according to the Environmental Noise Directive, environmental noise is described as unwanted or harmful sound derived from human activities, including noise emitted by means of transport — road traffic, rail traffic, air traffic, and from sites of industrial activity (Directive 2002/49/EC 2002). This directive defines day-evening-night noise levels above 55 dB(A) as harmful, with road traffic noise as the predominant source. More than 113 million people are affected by road traffic noise exposure above the recommended values, meaning that at least 20% of Europeans are exposed to traffic noise levels that can cause adverse health effects. The overall number of people exposed to noise levels above 55 dB originated by other means is 22 million for railway noise, 4 million for aircraft noise, and less than 1 million for noise created by industrial activities (WHO 2018).

According to several reviews conducted by some WHO expert chairs, exposure to road traffic noise could cause non-auditory health effects, including adverse birth outcomes (Nieuwenhuijsen et al. 2017), cardiovascular disease (CVD) and metabolic effects (van Kempen et al. 2018), sleep disturbances (Basner and McGuire 2018), or cognitive impairment (Clark and Paunovic 2018a). However, the evidence regarding the relationship between environmental noise and some of the mentioned outcomes is very scarce. This does not mean that there is no relationship, but more quality research is needed. Moreover, in Europe, long-term exposure to noise causes 12,000 premature deaths and 48,000 cases of ischemic heart disease per year. Furthermore, 6.5 million people experience chronic sleep disturbances, and 12,500 schoolchildren struggle with learning difficulties (European Environment Agency 2020).

In this review, we summarize the current understanding of the molecular pathways and mechanisms underlying the non-auditory health effects of noise. Our objective is to understand how noise cotributes in the most common health outcomes, including inflammation and oxidative stress, vascular dysfunction, dysregulation of the circadian rhythm, metabolic disturbances, age-related diseases, changes in the microbiome, and mental health outcomes. Most reviews addressing the mechanisms underlying noise-induced non-auditory effects have focused on only one health outcome, the most common being vascular dysfunction. However, given the interrelation between these outcomes, this review reinforces the importance of a holistic approach, considering all outcomes and their interactions.

## Noise reaction model

According to the noise reaction model proposed by Babisch (Babisch 2003), noise can induce harmful effects through two pathways. The model suggests that high noise levels (> 85 dBA), can directly cause health problems such as hearing loss or direct physiological changes due to sleep disturbances. In contrast, indirect pathway is related to lower noise levels impairing daily activities, communication or sleep. This pathway involves cognitive perception, leading to cortical activation and emotional responses like annoyance (Münzel et al. 2021). It is believed that when annoyance is high and chronic, a mechanism of psychological habituation occurs: isolation of noise from consciousness and reduction of emotional overload in the prefrontal cortex, resulting in less annoyance (Recio et al. 2016).

However, the physiological response to noise persists: It causes a primary stress reaction. Specifically, it triggers the activation of the hypothalamic–pituitary–adrenal (HPA) axis and the sympathetic nervous system. Hence, a cascade of reactions occurs, including the release of stress hormones such as cortisol, adrenaline, and noradrenaline(Daiber et al. 2019). HPA axis activity is governed by three hormones: corticotropin-releasing hormone (CRH), adrenocorticotropic hormone (ACTH), and cortisol, the main glucocorticoid in humans. Activation of the HPA axis triggers the release of CRH, which stimulates ACTH production and modulates cortisol synthesis. Elevated cortisol levels serve as a negative feedback mechanism, suppressing the release of CRH and ACTH and thus restoring basal levels of stress hormones (Herman et al. 2016). However, in cases of chronic stress, dysfunction in the HPA axis occurs, and buffering mechanisms may prove insufficient to return to baseline conditions. This physiological phenomenon is known as allostatic load and has been associated with several detrimental health outcomes (Mc Ewen 1998; Guidi et al. 2021). Noise has been identified as a chronic stressor that triggers a chain reaction of oxidative, inflammatory, and metabolic effects, resulting in non-auditory health outcomes (Hahad et al. 2021). A study that associates night-time aircraft noise exposure with an increased risk of Takotsubo syndrome, a cardiomyopathy linked to excessive stress hormone release, supports this idea of the importance of the indirect pathway (Münzel et al. 2016).

Cortisol measurement provides an estimation of HPA axis activity. Acute cortisol levels can be measured in biological samples, namely blood, saliva, and urine samples (Hellhammer et al. 2009; Wright et al. 2015; Mlili et al. 2021). Most of the research has focused on the effect of aircraft noise in salivary cortisol, reporting elevated salivary cortisol levels in participants living near airports (Selander et al. 2009; Lefèvre et al. 2017; Baudin et al. 2019). In contrast, studies in regard of road traffic noise are inconclusive. A systematic review concluded that road traffic noise was related to higher urinary or salivary cortisol levels (Hohmann et al. 2013); however, recent studies have shown no association between road traffic noise and salivary cortisol (Wallas et al. 2018; Bloemsma et al. 2021). Concerning chronic cortisol levels, hair cortisol was reported as a viable tool for assessing the link between environmental noise exposure and chronic stress (Michaud et al. 2022). To the best of our knowledge, only one study measured hair cortisol and found no association between residential exposure to road traffic noise and hair cortisol concentration in 14–15-year-old adolescents (Verheyen et al. 2021).

**Fig. 1:**
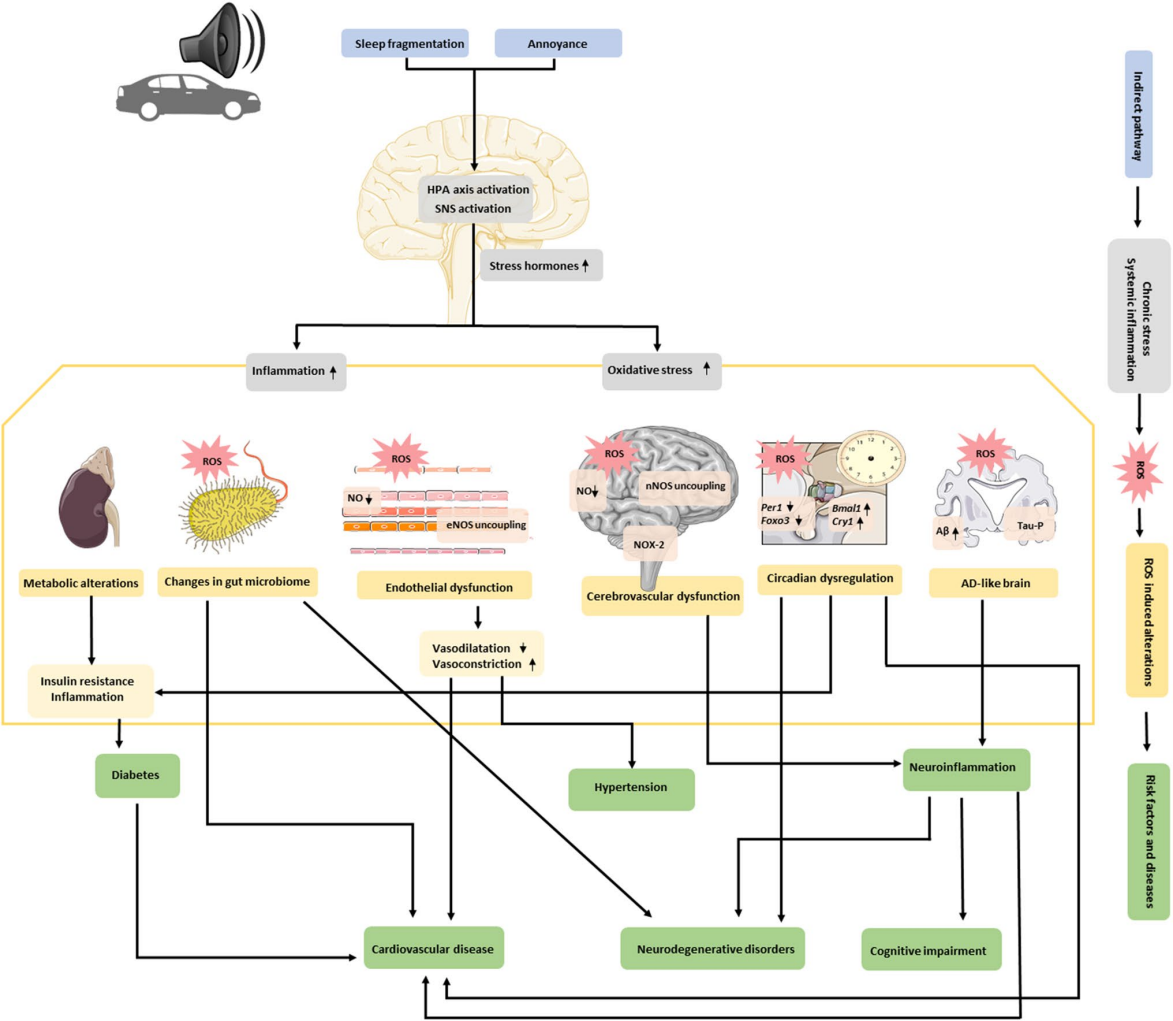
Summary of the current knowledge of the mechanism by which road traffic noise induces chronic stress hormones, systemic inflammation, and oxidative stress impact on several health outcomes. As proposed by the noise reaction model, in the indirect pathway, noise causes sleep disturbances and annoyance (represented in the upper side in blue), which cause HPA axis and SNS activation: higher levels of cortisol, systemic inflammation, and oxidative stress (in gray) cause the main detrimental health outcomes induced by reactive oxygen species (ROS) (summarized in yellow). Changes in the microbiome, noise-induced vascular dysfunction, neuroinflammation, dysregulation of the circadian rhythm, accelerated aging, and Alzheimer’s disease (AD) like brain, as well as the interrelationships among all of these health outcomes, result in the accumulation of multiple risk factors leading to serious adverse health effects (below in green*).* Figure made by author, based on the previous research

Therefore, it is thought that chronic release of stress hormones due to noise-induced activation of the HPA axis and sympathetic nervous system produces a state of chronic inflammation and oxidative stress, as detailed in Fig. 1

## Inflammation and oxidative stress

Inflammation is the body’s reaction through which immune and non-immune cells are activated, to eradicate harmful stimuli and promote tissue repair and recovery. An important aspect of the inflammatory response is temporal regulation: It is activated when a threat is present and ends once the threat is over (Furman et al. 2019). Factors that induce inflammation (e.g., pathogens, damaged cells, toxic chemicals, and physical and psychological stresses) trigger the production of inflammatory mediators. These mediators activate the downstream components of the inflammatory pathway (Medzhitov 2008; Hahad et al. 2019). This process is regulated and usually lasts for a few days, allowing elimination of the the threat without causing undue tissue damage (Leiba et al. 2023). Any failure in this control could provoke chronic inflammation, characterized by the infiltration of mononuclear immune cells (monocytes, macrophages, lymphocytes, and plasma cells), tissue destruction, and fibrosis (Khansari et al. 2009). While acute inflammation is vital for immune response, systemic chronic inflammation (SCI) has been linked to various diseases such as CVD, cancer, metabolic dysregulation, and neurodegenerative diseases (Furman et al. 2019).

The harmful effects of chronic inflammation are mainly caused by overproduction of reactive oxygen species (ROS) and depletion of antioxidants (Halliwell 2006). ROS, unstable molecular species with one or more unpaired electrons, are crucial for the regulation of several signalling pathways (e.g. cell differentiation, proliferation and antioxidant regulation) (Halliwell and Gutteridge 1985). Nonetheless, an imbalance between the production of ROS and antioxidant defenses, known as oxidative stress, can lead to a toxic increase in ROS levels, which can induce cell damage. Elevated ROS levels can generate other ROS, such as hydrogen peroxide (H_2_O_2_), superoxide anions (O2•^−^), and hydroxyl radicals (OH•). This, in turn, results in oxidative damage to cellular lipids and proteins and mutations in the genome, ultimately leading to cell death (Halliwell and Gutteridge 1985; Hajam et al. 2022). ROS-induced oxidative stress disrupts various organ systems, including the nervous system, kidneys, liver, and cardiovascular system (Khansari et al. 2009).

Several molecular pathways are activated in response to stress, leading to the excessive generation of ROS and inflammatory signalling. Myeloid cells trigger the initial response in the inflammatory process. Once recruited to the injury site, these cells generate ROS, as well as inflammatory cytokines, chemokines, and prostaglandins. Nuclear factor-κB (NF-κB), mitogen-activated protein kinases (MAPK) and JAK-STAT signalling pathways (Chen et al. 2018), and the transcription factor nuclear factor erythroid 2-related factor 2 (NRF2) inhibit cell death by promoting anti-inflammatory and antioxidant processes. NRF2 appears to serve as a vital protective mechanism against various environmental stressors (Bayo Jimenez et al. 2022). Noise-induced oxidative stress appears to activate NRF2 and trigger the production of its target genes. Conversely, NRF2 deficiency exacerbates noise-induced damage, while its activation has protective effects (Bayo-Jimenez et al. 2021).

## Inflammation and oxidative stress by road traffic noise exposure

Previous studies suggest that inflammation and oxidative stress play key roles in the development of by road traffic noise-induced damage (Daiber et al. 2020). The mechanisms through which it contributes to the overall disease burden remain unclear, primarily due to the absence of well-established research models in both humans and animals. Previous reviews have focused on the role of ROS and inflammation in noise-induced cardiovascular dysfunction (Daiber et al. 2019, 2020; Münzel et al. 2022), dysregulation of the circadian clock (Daiber et al. 2022), neurodegenerative disorders (Manukyan 2022), accelerated aging (Hahad et al. 2021), and psychiatric disorders (Hahad et al. 2022). However, we aim to provide a general overview of this issue and explore the interconnection between the diverse non-auditory health outcomes.

### Animal studies

Experimental research conducted on animal models has shown that noise exposure can lead to extra-auditory effects, mostly in the brain and immune system, by triggering oxidative stress (Cheng et al. 2011; Cui and Li 2013; Molina et al. 2016; Pascuan et al. 2014). Manikandan et al. (2006) reported heightened activity of antioxidant enzymes in the hippocampus of rats exposed to acute noise, whereas the activity decreased in those chronically exposed. In another study, mice exhibited increased immune function after 3-day noise exposure, but decreased immune function and oxidative stress were observed in mice exposed for 28 days (Zheng and Ariizumi 2007).

Münzel et al. established a protocol for aircraft noise exposure to study its effects on mice. They exposed mice to high levels of noise (maximum 85 dB, mean 72 dB) for 43 s, and repeated this exposure 69 times, trying to mimic aircraft noise exposure. This has been fully explained elsewhere (Münzel and Daiber 2018). Following this protocol for four consecutive days, they observed elevated systolic blood pressure, as well as increased levels of catecholamines, angiotensin-II, and endothelin-1. Noise-exposed animals exhibited signs of oxidative stress and inflammation, including eNOS uncoupling as well as increased levels of IL-6, expression of the NADPH oxidase 2 (NOX-2) protein, and nitrotyrosine-positive proteins. Additionally, they observed an increase in the infiltration of natural killer cells and neutrophils into the vasculature (Münzel et al. 2017). Neuroinflammation, cerebral oxidative stress, or circadian dysregulation due to aircraft noise exposure were avoided in *Nox2* knockout mice (Kröller-Schön et al. 2018), as well as the pro-inflammatory phenotype and activation of circulating leukocytes (Eckrich et al. 2021). Notably, same noise exposure protocol was used in the aforementioned studies. Frenis et al. found that elimination of monocytes and macrophages (the main lysozyme M-positive inflammatory cells) blocked noise-induced inflammation, oxidative stress, and vascular dysfunction, suggesting the relevance of NOX-2 to noise effects (Frenis et al. 2021a). Given the cross-activation of endothelin-1 and NOX-2, and the fact that both increase under noise exposure, the stimulation of one of them may lead to a vicious cycle that results in oxidative stress (the pathway is fully explained in Frenis et al. (2021b)). It should be noted that the majority of studies investigating noise-induced redox imbalance in animals used extremely high sound pressure levels, which try to mimic aircraft noise exposure rather than road traffic noise (Molina et al. 2016), and both loudness and other characteristics (frequency and pattern) may determine detrimental noise effects (Münzel et al. 2017). Moreover, animal studies are not always reliable predictors of human outcomes (Bracken 2009)."

### Human studies

Evidence from human field studies also suggests that oxidative stress plays an important role in noise-derived health effects. The administration of the antioxidant Vitamin C diminished endothelial dysfunction associated with train and aircraft noise exposure (Schmidt et al. 2013; Herzog et al. 2019). In a study published in 2020, the authors reported heightened activity in the amygdala among individuals residing in areas with high road traffic noise (Osborne et al. 2020). The amygdala, a part of the limbic system, is responsible for emotional responses, including fear, anxiety, and aggression. It also processes physiological and behavioural reactions to stress and plays a crucial role in the brain’s response to environmental stressors including noise (Spreng 2000; Powell-Wiley et al. 2021). Increased amygdalar activity is linked to a higher risk of CVD due to increased atherosclerotic inflammation (Osborne et al. 2022). In fact, higher noise exposure predicts major adverse cardiovascular events (MACE) and is associated with increased arterial inflammation (Osborne et al. 2020). Recently, the same researchers demonstrated that the combination of air pollution and road traffic noise also contributes to an increased risk of MACE and arterial inflammation (Osborne et al. 2022).

According to observational cohort studies, long-term exposure to road traffic noise induces alterations in blood biochemistry and immune response in adults, including elevated levels of IL-12 and high-sensitivity C-reactive protein (hsCRP) or a reduced NKT cell population (Cai et al. 2017; Kim et al. 2017; Kupcikova et al. 2021). However, it is worth noting that these findings were not uniform across all studies (Michaud et al. 2022). Interestingly, the Swiss SAPALDIA cohort concluded that DNA methylation was associated with long-term exposure to road traffic noise and air pollution. This association was linked to pathways related to inflammation, cellular development, and immune responses (Eze et al. 2020).

As previously noted, ROS are vital for the regulation of several signalling pathways that are linked to numerous health effects. Hence, the upcoming section provides a comprehensive overview of how inflammation and oxidative stress act as mediators of non-auditory health outcomes induced by road traffic noise.

## Noise-induced health effects

The state of chronic stress resulting from road traffic noise exposure, characterized by alterations in the HPA axis and stress hormones, systemic inflammation, and oxidative stress, can affect various systems and contribute to a wide range on non-auditory diseases (Fig. 1**)**. In this review, we focus on its effects on the circadian dysregulation, metabolic alterations, aging and age-related diseases, changes in the gut microbiome, vascular dysfunction, and mental health outcomes. Summary of the main findings explained in this section is available in Table 1, where both animal and human studies are presented.

**Table 1:** Summary of the main findings regarding the noise-induced non-auditory health outcomes

Health outcome	Main finding		Human/animal model	Noise exposure	Reference
Stress hormones					
		Increased salivary or urinary cortisol levels in participants under higher road traffic noise exposure	Human	Road traffic noise	Hohmann et al. (2013)
		No association between road traffic noise and salivary cortisol	Human	Road traffic noise	Wallas et al. (2018) Bloemsma et al. (2021)
		No association between residential exposure to road traffic noise and hair cortisol concentration	Human	Road traffic noise	Verheyen et al. (2021)
Inflammation					
		Increased immune function was observed after 3-day noise exposure and decreased immune function after 28 days of exposure	Mice	90 dB, 5 h/day, 3 or 28 days	Zheng and Ariizumi (2007)
		Increased levels of IL-6	Mice	85 dB during 43 s, 69 repetitions	Münzel et al. (2017)
		Pro-inflammatory phenotype and the activation of circulating leukocytes were avoided in Nox2 knockout mice	Mice	85 dB during 43 s, 69 repetitions	Eckrich et al. (2021)
		Exposure to road traffic noise causes increased levels of IL-12 and high-sensitivity C-reactive protein (hsCRP) levels, and decreased NKT cell population	Human	Road traffic noise	Cai et al. (2017); Kim et al. (2017); Kupcikova et al. (2021)
		Long-term exposure to road traffic noise and air pollution was associated with the enrichment of pathways related to inflammation, cellular development, and immune responses	Human	Road traffic noise	Eze et al. (2020)
Oxidative stress					
		Increased hippocampal activity of antioxidant enzymes in animals exposed to acute noise, while activity was decreased in chronically exposed ones	Rats	100 dB, 4 h/day, 30 days	Manikandan et al. (2006)
		Increased expression of NOX-2	Mice	85 dB during 43 s, 69 repetitions	Münzel et al. (2017)
		Neuroinflammation, cerebral oxidative stress, or circadian dysregulation due to aircraft noise exposure were avoided in *Nox2* knockout mice	Mice	85 dB during 43 s, 69 repetitions	Kröller-Schön et al. (2018)
		Antioxidant Vitamin C diminished noise-exposure induced endothelial dysfunction	Human	Train noise Aircraft noise	Herzog et al. (2019); Schmidt et al. (2013)
Metabolic alteration					
Noise causes metabolic alterations, induces alterations in blood biochemistry, diabetes, and insulin resistance			Mice	95 dB, 4 h/day, 20 days 400–3600 Hz (dB not defined)	Liu et al. (2016, 2018); Morakinyo et al. (2019)
Changes in gut microbiome					
		Probiotic treatment alleviated anxiety-like behaviour in noise exposed rats, by restoring functioning of HPA axis and gut-brain-microbiota axes	Rats	95 dB, 4 h/day, 30 days	Hadizadeh et al. (2019)
		Promoting gut microbiota homeostasis with Lactobacillus rhamnosus GG improved gut bacterial balance	Rats	20–20,000 Hz ( dB not defined)	Li et al. (2023a, b)
		Decreased gut microbiota diversity and compositional alterations were observed after noise exposure for 4 h/d during 30 consecutive days	Mice	400–3600 Hz (dB not defined)	Cui et al. (2016, 2018)
		Imbalance between oxidative and anti-oxidant pathways and systemic inflammation in response to noise were related with alterations in gut microbiota	Mice	20–20 kHz (98 dB, 4 h/day, 30 days)	Chi et al. (2021)
Vascular dysfunction *Cardiovascular dysfunction*					
		Exposure to aircraft noise caused endothelial dysfunction, increased blood pressure, increased levels of neurohormones, and higher sensitivity to vasoconstrictors, including endothelin-1 and noradrenalin. Reduction in NO bioavailability was observed, due to eNOS uncoupling/dysfunction and NO reaction with superoxide in an oxidative state	Mice	85 dB during 43 s, 69 repetitions	Kröller-Schön et al. (2018); Münzel et al. (2017)
		Offsetting upregulation eNOS mRNA levels and main enzymes responsible for eNOS cofactor	Mice	85 dB during 43 s, 69 repetitions	Münzel et al. (2017)
		Road traffic noise exposure caused endothelial dysfunction markers of inflammation and oxidative stress	Human	Train noise Aircraft noise Aircraft noise	Herzog et al. (2019); Schmidt et al. (2015); Schmidt et al. (2013)
*Cerebrovascular dysfunction*					
		Reduced dendritic count in the hippocampus and increased oxidative stress in the frontal cortex after exposure to noise	Rats	100 dB, 4 h/day, 30 days	Manikandan et al. (2006)
		Exposure to noise caused hippocampal alterations that could underlie behavioural effects	Rats	95–97 dB, 2 h/day, Single day/five consecutive days	Uran et al. (2012)
		Noise-induced hippocampal oxidative stress and changes in aminoacidergic neurotransmitters	Rats	95–97 dB, 2 h/day, Single day/15 consecutive days	Molina et al. (2020)
		Downregulation and malfunction of nNOS results in NO bioavailability reduction, which in turn causes neuroinflammation, downregulation of FOXO3, and oxidative stress	Mice	85 dB during 43 s, 69 repetitions	Kröller-Schön et al. (2018)
		α2-adrenoblockers reduce noise-induced oxidative stress and cognitive damage	Rats	91 dBA, 8 h/day, 60 days	Melkonyan et al. (2021)
		Reduced dendritic count in the hippocampus and increased oxidative stress in the frontal cortex after exposure to noise	Rats	100 dB, 4 h/day, 30 days	Manikandan et al. (2006)
Circadian dysregulation					
		More than 30 circadian gene expressions were altered: reduction in the expression of *Per1* and *Foxo3* and upregulation of *Bmal1* and *Cry1*	Mice	85 dB during 43 s, 69 repetitions	Kröller-Schön et al. (2018)
		Circadian rhythm disturbances in people exposed to night-time road traffic noise	Human	Road traffic noise	Eze et al. (2017)
Aging and age-related diseases					
Overproduction of Aβ and hippocampus neuroinflammation in rats after noise exposure			Rats	400–3600 Hz (dB not defined)	Cui et al. (2015)
		30 days of noise exposure caused increased tau phosphorylation in the hippocampus	Rats	95 dB, 4 h/day, 30 days	Gai et al. (2017)
		Chronic noise exposure caused overproduction of Aβ and increased the hyperphosphorylation of tau in the hippocampus and prefrontal cortex (PFC) in young SAMP8 mice	Mice	400–3600 Hz (dB not defined)	Su et al. (2018)
		No association between noise subdomain and telomere length	Human	Perceived road traffic noise	Park et al. (2015)

### Noise and metabolic alterations

Road traffic noise has been suggested to alter metabolic homeostasis and is mainly associated with diabetes mellitus. As previously stated, noise induces changes in the HPA axis, prompting the release of cortisol and other stress hormones, potentially resulting in metabolic disturbances (Babisch 2003). Metabolic alterations are also positively correlated with sleep disorders and circadian disruptions (Depner et al. 2014; Smiley et al. 2019), and these alterations have been associated with noise (Basner and McGuire 2018).

#### Human studies

A WHO expert review found that there is still a limited number of publications exploring the connection between road traffic noise and metabolic changes, and the existing results are inconsistent. Therefore, evidence for the risk of getting type-2 diabetes mellitus in response to traffic noise is of low quality (van Kempen et al. 2018). However, more recent studies have observed a positive association between the risk of type-2 diabetes mellitus development and road traffic noise (Ohlwein et al. 2019; Liu et al. 2023), and even long-term exposure to combined noise sources (Sørensen et al. 2023).

#### Animal studies

Animal studies also support the idea that noise induces metabolic alterations, with several studies showing that noise exposure provokes alterations in blood biochemistry, diabetes, and insulin resistance in mice (Liu et al. 2016; Morakinyo et al. 2019). Notably, in these experiments, high-noise exposures were used, which may be far from real road traffic noise exposure characteristics. Table 1 resumes noise exposure characteristics used in animal studies: high intensity (around 85–100 dB) noises, exposed during a certain time, repeated several times. Road traffic noise, however, is usually more constant and with lower intensity. It has to be noted that road traffic noise levels could reach even higher levels in some cities, most of them Asian cities (United Nations 2022). Thus, noise exposures used during animal experiments are similar to aircraft noise: high intensity, intermitted noises, usually separated by a noise-free period (Basner et al. 2017). Although evidence regarding the link between road traffic noise exposure, metabolic alterations, and type-2 diabetes mellitus remains unclear, existing studies imply a potential mechanistic connection involving annoyance, sleep disturbances, alterations in the HPA axis, and the release of stress hormones. Noise could impact glucose metabolism by promoting liver glucose production, decreasing glucose absorption, encouraging fat breakdown in adipocytes, and inhibiting insulin secretion. These effects can result in insulin resistance and inflammation, both linked to diabetes development (Sharma and Singh 2020). In addition, metabolic alterations, abnormal lipid profiles, and insulin resistance are risk factors for CVD (Ormazabal et al. 2018).

### Noise and microbiome

In recent years, there has been a significant surge in research investigating the link between gut microbiota and various diseases. Furthermore, the relationship between inflammation, redox signaling, and the gastrointestinal microbiome has been elucidated (Frenis et al. 2021b). Stress has the ability to influence these gastro-intestinal microorganisms, as circulating concentrations of glucocorticoids and catecholamines can modulate microbial growth (Karl et al. 2018). It has to be noted that changes in gut microbiota are associated with several diseases, such as cardiometabolic diseases, neuroinflammation, and neurodegenerative disorders (Collins et al. 2012; Jones and Neish 2017; Campbell and Colgan 2019; Mou et al. 2022), which are also associated with road traffic noise (Münzel et al. 2021; Hahad et al. 2022).

#### Animal studies

Although few studies have focused on noise exposure and the microbiome, decreased gut microbiota diversity and compositional alterations were observed in mice exposed to high-noise for 4 h per day for 30 consecutive days (Cui et al. 2016, 2018). Another study discovered that disruptions in the gut microbiota were associated with an imbalance between oxidative and anti-oxidant pathways, reduced tight junction protein levels in the intestine and hippocampus, and systemic inflammation triggered by noise (Chi et al. 2021). Moreover, a recent study observed that the imbalance of microbiota–gut–brain axis in reponse to noise exposure differed between male and female rats (Li et al. 2023a, b). Hadizadeh et al. observed that administering probiotics to noise-exposed rats alleviated anxiety-like behaviour by restoring the proper functioning of the HPA axis and the gut-brain-microbiota connections (Hadizadeh et al. 2019). In addition, promoting gut microbiota homeostasis with Lactobacillus rhamnosus GG improved gut bacterial balance in noise exposed rats (Li et al. 2023a, b). Although more studies are needed, noise exposure may disrupt gut-brain-axis, leading to microbiota alterations, which exacerbates inflammation and oxidative stress and increases the risk of other diseases (Fig. 1). As far as we know, all studies has been conducted in animal models and this may not reflect human response to road traffic noise exposure.

### Noise and vascular dysfunction

#### Cardiovascular dysfunction

##### Human studies

Cardiovascular diseases, including ischemic heart disease (IHD), heart failure and arrhythmia, stroke, and arterial hypertension, stand as the primary causes of most noncommunicable diseases, accounting for 70% of global deaths (WHO 2011). The association between CVD risk and road traffic noise exposure has been widely studied (Babisch et al. 1988). In 2018, a comprehensive review conducted by a WHO expert panel established a strong link between road traffic noise and IHD, supported by high-quality evidence. A pooled analysis of seven longitudinal studies showed that for each 10 dB increase in road traffic noise, there was an increase of 1.08 in the relative risk (RR) (95% CI 1.01–1.15) for having an IHD. The quality of evidence for other noise sources or outcomes (e.g., stroke and arterial hypertension) was rated from moderate to very low (van Kempen et al. 2018), although recent studies have suggested that aircraft noise is mostly associated with CVD risk (Pyko et al. 2023; Saucy et al. 2021; Yankoty et al. 2021; Vienneau et al. 2022). In addition, despite the existence of over 35 cross-sectional publications examining the relationship between road traffic noise and hypertension, their quality was rated as “very low,” since most of them were cross-sectional studies(van Kempen et al. 2018). In the case of road traffic noise and stroke, the WHO review concluded that it was of moderate quality, with only one study showing a higher risk of stroke (van Kempen et al. 2018). Nevertheless, recent publications have surfaced since 2018. Among these, some indicated a heightened risk of stroke (Halonen et al. 2015; Roswall et al. 2021; Seidler et al. 2018; Sørensen et al. 2021), while the remaining two found no association (Hansell et al. 2018; Pyko et al. 2019). A recent review concluded that in the last years several high-quality cohort studies consistently found road traffic noise to be associated with a higher risk of ischemic heart disease, heart failure, diabetes, and all-cause mortality (Sørensen et al. 2024). It is important to note that road traffic noise could also be linked to other outcomes that are considered cardiovascular risk factors, such as sleep disturbances and circadian alterations, diabetes mellitus, annoyance, obesity, age-related diseases, changes in the microbiome, or poorer mental health (Münzel et al. 2023).

##### Animal studies

As previously stated, elevated NOX-2 levels were detected in the vasculature of mice exposed to aircraft-like noise, leading to ROS formation and endothelial dysfunction (Münzel et al. 2017). The reduction of noise-induced oxidative stress in *Nox2* knockout mice highly suggests the involvement of inflammatory cell-derived ROS (Kröller-Schön et al. 2018). In addition, oxidative stress, in turn, induces endothelial dysfunction. Mice exposed to aircraft noise present endothelial dysfunction, increased blood pressure, increased levels of neurohormones, and higher sensitivity to vasoconstrictors, including endothelin-1 and noradrenalin (Münzel et al. 2017; Kröller-Schön et al. 2018). These findings were extended to human studies, in which endothelial dysfunction was mediated by impaired sleep and/or higher levels of stress hormones, markers of inflammation, and oxidative stress after train and aircraft noise exposure (Schmidt et al. 2013, 2015; Kröller-Schön et al. 2018; Herzog et al. 2019). To the best of our knowledge, no studies have focused on road traffic noise.

Endothelial dysfunction caused by noise is related to the malfunctioning/uncoupling of endothelial nitric oxide synthase (eNOS) (Münzel et al. 2017; Kröller-Schön et al. 2018). eNOS synthesizes nitric oxide (NO) in the endothelium, an important protective molecule in the vascular system. The proper functioning of eNOS relies on the enzyme-forming dimers and the presence of the essential cofactor BH4 (Förstermann and Münzel 2006). Münzel et al. observed a reduction in vascular NO bioavailability in the aorta of high noise-exposed mice, which could explain the noise-induced endothelial dysfunction (Münzel et al. 2017). The reduction of NO bioavailability might result from oxidative stress induced by noise, which produces a highly reactive superoxide (O_2_^−^) intermediate. This intermediate reacts with NO to form peroxynitrite (ONOO^−^) leading to eNOS uncoupling (Förstermann and Münzel 2006; Daiber et al. 2020). In addition, in an oxidative stress state, BH4 is oxidized causing eNOS dysfunction (Landmesser et al. 2003; Schulz et al. 2008). In noise-exposed mice, an increase in the expression of key enzymes involved in BH4 synthesis (GTP-cyclo-hydrolase-1 and dihydrofolate reductase) was noted. This indicates a compensatory pathway to overcome eNOS uncoupling and endothelial dysfunction (Münzel et al. 2017). In addition, eNOS uncoupling could also be explained by eNOS S-glutathyionylation and phosphorylation in the presence of oxidative stress (Münzel et al. 2017; Kröller-Schön et al. 2018; Frenis et al. 2021b).

In summary, in response to noise, there is a reduction in NO bioavailability (due to eNOS uncoupling/dysfunction and NO reaction with superoxide in an oxidative state) and an increased sensitivity of blood vessels to vasoconstrictors, such as noradrenaline, endothelin-1, and angiotensin II (Münzel et al. 2018). Interestingly, heme oxygenase-1 (HO-1) and NRF2 induction improves noise-induced damage in aircraft-like noise-exposed mice (Bayo-Jimenez et al. 2021). Although more epidemiological and longitudinal studies are needed regarding road traffic noise effects on CVD, eNOS involvement in noise-induced endothelial dysfunction seems clear (Fig. 1). Furthermore, circadian dysregulations, metabolic alterations, aging, and changes in the gut microbiome are also considered risk factors for the development of CVD.

#### Cerebrovascular dysfunction

##### Human studies

According to the WHO, more than 12,500 children suffer from learning impairments at school (WHO 2018). A systematic analysis performed by the WHO concluded that there was substantial evidence for the association between aircraft noise and cognitive impairment in children, with a higher quality of evidence for the detrimental effect of aircraft noise on children’s reading and oral comprehension. Nonetheless, evidence regarding road traffic noise and cognitive development is still limited, due to the cross-sectional nature of most studies (Clark and Paunovic 2018a).

In addition, several studies have examined the impact of environmental noise on neurodevelopmental disorders. A meta-analysis deduced an increased odds ratio (OR) of hyperactivity/inattention (OR 1.11, 95% CI 1.04–1.19) and total difficulties (OR 1.09, 95% CI 1.02–1.16) per 10 dB increase in road traffic noise exposure in children (Schubert et al. 2019). In contrast, Essers et al. observed no association between exposure to road traffic noise during prenatal and childhood periods and emotional, aggressive, or attention-deficit/hyperactivity disorder (ADHD)–related symptoms, in children from two European birth cohorts (Essers et al. 2022).

##### Animal studies

The effect of noise on cognition is supported by animal experiments, although experimental noise exposure may not reflect road traffic noise exposure. These studies demonstrated anatomical changes in neurons, several hippocampal alterations, and increased oxidative stress in the frontal cortex and hippocampus of rats after noise exposure. Additionally, noise exposure also led to cognitive and memory impairment and deterioration of motor coordination (Manikandan et al. 2006; Uran et al. 2012; Kröller-Schön et al. 2018; Jafari et al. 2020; Molina et al. 2020). Molina and Guelman (2022) explain that the hippocampus is particularly vulnerable to noise-induced damage and is even more susceptible than the auditory cortex (Cheng et al. 2016). It is important to note that noise could cause hippocampal-dependent behavioural changes in rats (Molina et al. 2022; Uran et al. 2012), and some noise-induced behavioural changes, such as sleep disturbances, can cause alterations in the hippocampus (Molina and Guelman 2022). Another investigation showed that noise can induce changes in neurotransmitter release, long-term potential and synaptic plasticity in mice (Metz et al. 2001). In those experiments, animals were also exposed to high intensity aircraft-like noises. Table 1 presents more information regarding noise exposure characteristics.

While the precise mechanisms behind the cerebral effects of noise remain uncertain, it appears that the activity and expression of neuronal nitric oxide synthase (nNOS) protein play a role. nNOS produces NO, which displays many properties of a neurotransmitter in the brain and in the peripheral nervous system. Noise exposure in mice leads to decreased expression of the protein and in its uncoupling in cerebral tissue (Kröller-Schön et al. 2018). Specifically, phosphorylation mediated by redox-sensitive calcium/calmodulin-dependent protein kinases causes its inactivation and uncoupling (Kasamatsu et al. 2014). Furthermore, the application of the targeted nNOS inhibitor ARL-17477 enabled the identification of uncoupled nNOS as the primary source of ROS in the cerebral tissue (Kröller-Schön et al. 2018).

Similar to vascular dysfunction, the reduction and impaired function of nNOS lead to diminished availability of NO. This deficiency triggers neuroinflammation, downregulation of FOXO3, and oxidative stress (Kröller-Schön et al. 2018). α2-adrenoblockers mitigate oxidative stress and cognitive damage caused by noise, suggesting their involvement in response to noise exposure (Melkonyan et al. 2021).

In summary, nNOS dysregulation, NOX-2 upregulation, and NOX-2 dependent ROS production (mainly in the hippocampus and prefrontal cortex) could explain the learning and memory impairment observed in mice (Kan et al. 2015), as well as the impaired cognitive development in children exposed to road traffic noise (Foraster et al. 2022), as presented in Fig. 1. Nonetheless, more longitudinal human studies are needed to disclose in more detail the mechanism.

#### Noise and mental health

##### Human studies

Noise is recognized to induce annoyance, sleep disturbances, and psychological stress. These factors might contribute to the impact of noise on an individual's quality of life, overall well-being, and mental health. Nonetheless, a systematic review published in 2018 concluded that there was very low-quality evidence across available studies regarding the effect of road traffic noise on depression, anxiety, and emotional disorders, given that many studies had small sample sizes and most of them were cross-sectional studies (Clark and Paunovic 2018b). In a 2020 update, the authors concluded that the quality of evidence linking environmental noise to mental health had improved since the previous review. The evidence was rated as low, whereas in the 2018 review, it was rated as very low (Clark et al. 2020). Recently, numerous studies have explored the influence of road traffic noise on mental illness. These studies have revealed positive links between road traffic noise and symptoms associated with anxiety, tension, depression, bipolar disorder (Hao et al. 2022), and incident psychological ill-health (Stansfeld et al. 2021). A recent study revealed an association between exposure to road traffic noise and risk of death by suicide (Wicki et al. 2023). More longitudinal studies investigating the standardized definitions of depression and anxiety are needed.

##### Animal studies

To the best of our knowledge, evidence regarding the mechanistic pathways linking road traffic noise exposure and mental health in humans is lacking. However, noise-induced changes in the brain tissue, circadian dysregulation, changes in the immune cells, and remote organ damage causing feedback signalling may play a key role in the development of mental health problems (Hahad et al. 2024). Noise has been shown to trigger changes in the central nervous system in rats and mice (Cui et al. 2015; Gai et al. 2017; Su et al. 2018). Considering road traffic noise as an environmental stressor, it may cause chronic stress. Chronic stress is associated with volume reductions in specific areas of the prefrontal cortex and limbic system, as well as alterations in neuronal plasticity. These changes may give rise to cognitive, emotional, and behavioural impairments, increasing the likelihood of developing psychiatric disorders. (Lucassen et al. 2014). The production of transcription factor regulated in development and DNA damage responses 1 (REDD1) in the presence of chronic stress plays a crucial role in the disruption of neuronal protein and neurotrophic factor synthesis, spine formation, and synaptic plasticity by altering mTORC1 signalling (Ota et al. 2014).

Moreover, the role of NO in the development of depression is vital, as it regulates neurotransmitters essential to this condition, including norepinephrine, serotonin, dopamine, and glutamate (Dhir and Kulkarni 2011). As mentioned previously, noise-induced reduction in vascular NO bioavailability in mice has been observed (Münzel et al. 2017). Furthermore, psychosocial stress and road traffic noise are thought to elevate inflammation biomarkers, including inflammatory cytokines (Haroon et al. 2012; Münzel et al. 2017). The connection between road traffic noise and mental health issues is reinforced by the concept that inflammatory cytokines mediate the cerebral neurochemical alterations that contribute to the onset of depression (Felger and Miller 2012; Haroon et al. 2012).

Although it seems that there is a pathway linking road traffic noise and mental health illnesses, more evidence is needed, from both epidemiological and cellular and molecular sudies.

### Noise and the circadian system

The circadian clock controls several essential biological functions including sleep, cognitive function, body temperature, and appetite. Circadian rhythms refer to biological cycles that occur within a 24-h period to synchronize physiological processes and adapt to day-night changes in the environment (Crnko et al. 2019). These rhythms are controlled by the central clock located in the suprachiasmatic nucleus of the hypothalamus. This central clock coordinates with peripheral clocks present in almost all mammalian tissues (Greco and Sassone-Corsi 2019). Responsive to various stimuli such as light, food, exercise, and social cues, the clock releases signaling molecules like cortisol or melatonin (Li et al. 2020). This clock operates under a tightly regulated transcriptional-translation feedback loop cycling every 24 h and depends on specific genes such as circadian locomotor output cycles protein kaput (*CLOCK*); periods 1, 2, and 3 (*PER1, PER2* and *PER3*); cryptochrome 1 and 2 (*CRY1* and *CRY2*); and brain and muscle arnt-like protein-1 (*BMAL1* and *BMAL2*). When CLOCK and BMAL1 form a complex, they attach to the promoters of *PER* and *CRY* starting transcription of those proteins. PER and CRY act as negative feedback, hindering *CLOCK* and *BMAL1* transcription, and thus, regulating its own transcription (Naito et al. 2003). The circadian clock can alter the cellular redox balance by activating genes related to redox production or antioxidants, like those controlled by NRF2. Likewise, redox balance influences the circadian rhythm (Merbitz-Zahradnik and Wolf 2015).

#### Human studies

There is ample evidence linking road traffic noise exposure and sleep disturbances, which provides indirect evidence linking road traffic noise exposure and circadian dysregulation (Daiber et al. 2022). Moreover, the indirect pathway proposed by Babisch highlights the chronic stress response caused by noise-induced sleep disturbances (Babisch 2003). A systematic review conducted by the WHO concluded that there is high-quality evidence to state that road traffic noise affects both objectively measured sleep physiology and subjectively assessed sleep (Daiber et al. 2022). Importantly, circadian misalignment is often seen in shift workers and the resulting disturbances in circadian rhythms have been linked to an increased risk of metabolic changes and CVD (Naito et al. 2003).

The Swiss Cohort Study SAPALDIA detected disruptions in circadian rhythms among individuals exposed to nighttime road traffic noise, indicating a relationship between road traffic noise at night and the circadian clock. They identified a strong interaction for melatonin receptor 1B rs10830963, an acknowledged diabetes risk variant linked to melatonin profile imbalance (Eze et al. 2017).

#### Animal studies

This relationship between the circadian clock and redox state is reciprocal. The cellular redox state can influence the circadian clock (Daiber et al. 2022) which can boost the expression of genes related to redox production or antioxidants (Naito et al. 2003). Dysregulation of redox regulation by circadian clock genes has been linked to several diseases such as CVD and diabetes (Young et al. 2002; Naito et al. 2003). Given the redox changes induced by noise in several tissues and the redox regulatory mechanisms of the circadian clock, exposure to road traffic noise may also affect the circadian clock. In mice exposed to noise similar to aircraft noise, over 30 circadian genes showed altered expression levels. Notably, there was a decrease in *Per1* and *Foxo3* expression accompanied by increased levels of *Bmal1* and *Cry1*. FOXO3 signalling appears to play a key role in this regulation. Intriguingly, these alterations were absent in mice exposed to noise during the awake phase (Kröller-Schön et al. 2018).

Although the whole mechanism has not yet been discovered yet, the effect of road traffic noise exposure on sleep disturbances and circadian rhythm dysregulation seems clear. Disturbances in circadian rhythm can also be associated with inflammation, metabolic alterations, CVD, and neuroinflammation (Young et al. 2002; Naito et al. 2003) (Fig. 1).

### Noise, aging, and age-related diseases

Aging is a natural and irreversible process marked by a gradual decline in physiological functions, ultimately resulting in age-related illnesses such as cardiovascular diseases, neurodegenerative disorders, and cancer (Li et al. 2021). A key feature of aging is the accumulation of senescent cells that release numerous inflammatory mediators. Genomic instability, reduced telomere length, epigenetic alterations, stem cell exhaustion, and mitochondrial dysfunction are the main hallmarks of ageing (López-Otín et al. 2013). The involvement of oxidative stress in several age-related conditions (Wickens 2001) implies that noise might also impact the aging process.

#### Human studies

Alzheimer’s disease (AD) is a common age-related disease, and environmental stressors are considered risk factors for its development (Manukyan 2022). Recent studies have shown that road traffic noise is associated with a higher risk for cognitive impairment, dementia, and AD (Fuks et al. 2019; Cantuaria et al. 2021; Weuve et al. 2021). However, other studies found no effect on dementia (Andersson et al. 2018; Carey et al. 2018). The NESDA cohort investigated the connection between neighborhood quality and telomere length, finding no association with the noise subdomain (Park et al. 2015). Furthermore, recent studies have focused on the impact of road traffic noise on cancer risk, concluding that it may increase the likelihood of developing cancer (Roswall et al. 2023; Sørensen et al. 2024; Thacher et al. 2023).

#### Animal studies

Human data on noise exposure and biological aging measurements such as telomere length and DNA methylation are almost missing, with most evidence on this matter resulting from animal studies (Guo et al. 2017; Dorado-Correa et al. 2018). Cui et al. observed that noise exposure accelerated the overproduction of amyloid β (Aβ) and the amyloid precursor protein along with inducing hippocampal neuroinflammation in rats. The authors proposed that noise elevates pro-inflammatory mediators and decreases anti-inflammatory factors. This imbalance leads to amyloid accumulation, mitigates neuroprotective effects, and exacerbates neuroinflammatory pathology (Cui et al. 2015) and may be associated with a disrupted autophagic flux homeostasis (Li et al. 2019; Zheng et al. 2022). Another study suggested that stress contributes to tau hyperphosphorylation, as increased tau phosphorylation and expression of corticotrophin releasing factor (CRF) were observed in the hippocampus of rats after 30 days of noise exposure (Gai et al. 2017). A more recent investigation found that chronic noise exposure led to excess production of Aβ and heightened hyperphosphorylation of tau in the hippocampus and prefrontal cortex (PFC) of young senescence-accelerated mouse prone 8 (SAMP8). This mouse model is particularly valuable for studying accelerated senescence linked to AD. The characteristics of young noise-exposed mice were similar to those of aged SAMP8 mice, and Wnt signalling was decreased in both cases, suggesting that inhibition of this pathway may help inducing aging (Su et al. 2018). This pathway is believed to be neuroprotective against Aβ toxicity and its deregulation may be involved in the pathogenesis of AD (Palomer et al. 2019). Again, aircraft-like noise was used in the aforementioned experiments. Furthermore, neurovascular dysfunction associated with noise exposure can trigger a cascade of reactions leading to neurodegeneration, thereby contributing to the progression of dementia and AD (Nelson et al 2016).

From a mechanistic point of view, more evidence is needed to answer questions regarding the relationship between road traffic noise, aging, and age-related diseases; however, the stress response followed by neuroinflammation and oxidative stress, definitely plays a crucial role. It is important to highlight that aging is also associated with the development of cardiovascular and neurodegenerative diseases, which are also related to road traffic noise exposure (Fig. 1).

## Conclusion and future directions

This review summarizes the current knowledge regarding the molecular pathways underlying main road traffic noise-induced non-auditory health effects. Although more studies in humans are needed, the chronic stress state, altered HPA axis activity and abnormal levels of stress hormones appear to be central in the development of noise-induced inflammation and oxidative stress. According to the model proposed in this article, chronic inflammation and redox state play a key role in the development of road traffic noise-induced health effects as they are related to circadian dysregulation, aging and age-related diseases, changes in the gut microbiome, vascular (endothelial and cerebrovascular) dysfunction, and neuroinflammation. Herein, we point out that the interdependent and additive effects of all proposed pathways contribute to the risk factors associated with the development of CVD, neurodevelopmental impairment, metabolic disorders, and neurodegenerative diseases related to environmental noise exposure.

Although the proposed model is plausible and grounded in scientific literature, further studies on the issue are needed. Firstly, animal experiments have focused on aircraft-like noise: short-term and extremely high sound pressure levels. Considering that loudness and other characteristics (frequency and pattern) may determine detrimental noise effects, it would be interesting to develop a protocol to study the long-term effects of road traffic noise; however, it is usually more constant and with lower intensity, excepting for some big polluted cities. Since animal studies do not always reliably predict human outcomes, studies regarding mechanistic insight should concentrate on human studies, specifically focusing on oxidative stress and inflammation markers. Furthermore, as mentioned before, the measurement of stress hormones in response to noise exposure, such as chronic cortisol levels, would also be useful for understanding noise-mediated stress responses. More epidemiological studies are required to assess the effects of road traffic noise on aging, age-related diseases, neurodegenerative diseases, and mental health problems. In this sense, longitudinal studies could be helpful in analysing the causal effects on these disorders.

Considering environmental noise is the second most serious environmental risk factor, it is crucial to focus on the mechanisms by which road traffic noise affects human health, as it is the primary source of noise pollution. Understanding the specific pathways related to noise exposure can aid in the development of strategies and public policies to mitigate its deleterious effects.

## Data Availability

As this is a bibliographic review of current literature, data was not used.

## Abbreviations

ACTH: Adrenocorticotropic hormone
AD: Alzheimer’s disease
AMPK: AMP-activated kinase
ADHD: Attention-deficit/hyperactivity disorder
BMAL1 and BMAL2: Brain and muscle arnt-like protein-1 and protein-2
CVD: Cardiovascular disease
CLOCK: Circadian locomotor output cycles protein kaput
CRH: Corticotrophin-releasing hormone
CRY1 and CRY2: Cryptochromes 1 and 2
eNOS: Endothelial nitric oxide synthase
HO-1: Heme oxygenase-1
HPA: Hypothalamic-pituitary-adrenal
IHD: Ischemic heart disease
MACE: Major adverse cardiovascular events
MAPK: Mitogen-activated protein kinases
NIH: National Institutes Health
nNOS: Neuronal nitric oxide synthase
NO: Nitric oxide
NF-κB: Nuclear factor-κB
NRF2: Nuclear factor erythroid 2-related factor 2
OR: Odds ratio
PER1, PER2, and PER3: Periods 1, 2, and 3
ROS: Reactive oxygen species
REDD1: Regulated in development and DNA damage responses 1
RR: Relative risk
SCI: Systemic chronic inflammation
NOX-2: NADPH oxidase
WHO: World Health Organization
